# The effect of unilateral cortical blindness on lane position and gaze behavior in a virtual reality steering task

**DOI:** 10.1038/s41598-026-35805-x

**Published:** 2026-02-28

**Authors:** Arianna P. Giguere, Matthew R. Cavanaugh, Krystel R. Huxlin, Duje Tadin, Brett R. Fajen, Gabriel J. Diaz

**Affiliations:** 1 Rochester Institute of Technology, Center for Imaging Science, Rochester, NY 14620 USA; 2 University of Rochester, Rochester, NY 14642 USA; 3 Rensselaer Polytechnic Institute, Troy, NY 12180 USA

**Keywords:** Cortical blindness, Motion processing, Virtual reality, Steering, Neuroscience, Psychology, Psychology

## Abstract

Adults with cortically-induced blindness (CB) affecting a quarter to a half of their visual field show greater variability in lane positioning when steering compared to those with intact vision. Because humans rely on visual information from optic flow to control steering, we hypothesized that these lane biases are caused in part by a disruption to motion processing caused by CB. To investigate, we examined the steering behavior of 21 CB drivers (11 left-sided, 10 right-sided visual deficits) and 9 visually intact controls in a closed-loop virtual reality steering task. Participants were instructed to maintain a central lane position while traveling at 19 m/s along a procedurally generated single-lane road. Turn direction (left/right) and turn radius (35m/55m/75m) varied between trials, and the quality of optic flow information was indirectly manipulated by altering the environmental texture density (low/medium/high). Right-sided CB participants maintained a similar average distance from the inner road edge as controls. Those with left-sided CB were less affected by changes in optic flow and turn direction. These differences were not explained by age, time since stroke, sparing of central vision, gaze direction, or saccade rate. Our results suggest that some left-sided CB participants place a lower weighting on optic flow information in the control of steering, possibly as a result of lateralization in the processing of motion. More broadly, our findings show that CB steering and gaze behavior are remarkably preserved despite the presence of visual deficits across large portions of the visual field.

## Introduction

Stroke is the main cause of damage to the primary visual cortex (V1) in humans, leading to cortically-induced blindness (CB) – a loss of conscious vision through both eyes^1,2^. Its incidence is high and increasing: the 2009 National Hospital Discharge Survey reported $\approx$1 million strokes in the US each year. CB deficits occur in 27-57% of strokes^1^, suggesting that up to half a million individuals in the US may be newly affected by CB annually, joining the several million patients already living with this condition. In at least 22 US states, CB patients are legally prohibited from driving^3^. Although this places great limitations on autonomy and overall quality of life^1,4,5^, many CB patients exhibit abnormalities in driving behavior, including poor lane positioning, steering stability, and impaired detection of potential hazards^6^. While there is considerable variability in driving skills^7–9^ and compensatory scanning behavior involving the eyes and head^6,8,10^, CB drivers experience significantly more motor vehicle accidents than visually-intact controls^11^. Overall, there is a pressing need to better understand driving behavior in CB. In the present study, we investigated whether differences in steering behavior exhibited by CB drivers are caused by a deficit in their ability to process the motion information that people rely on for steering. We posit that knowing how CB influences visual motion processing in driving contexts, has the potential to inform guidance for these patients, as well as vision rehabilitation efforts^12^.

Fundamentally, the control of heading direction while driving is done by steering, which is thought to be modulated by a variety of inputs, including visual features^13^, vestibular and proprioceptive signals^14^, and non-retinal feedback from eye movements^15,16^. Often, it is an optimal combination of these inputs^15,17,18^ that is used to perceive self-motion through the world and control steering. Of the many potential visual cues used for navigation control, the perceived pattern of motion caused by relative movement between an environment and an observer, or “optic flow”, has been found to play a primary role^19–21^. Optic flow was first described by Gibson in 1950^22^, who suggested that the location in the direction of an observer’s heading, called the focus of expansion (or FoE) of optic flow, could serve as an informative visual cue to instruct heading direction during navigation. Decades of research has supported the claim that optic flow is a source of information that plays a large role in heading selection and steering control^23–30^, and further research has provided evidence that direction of heading judgments are more accurate when optic flow cues are paired with consistent vestibular motion cues^18,31,32^ or the egocentric direction of the target^28,33^. Together, these observations support the notion that optic flow helps facilitate visually-guided navigation. A central question of this paper is whether unilateral damage to early visual cortex, as in CB, affects the processing of optic flow that guides steering behavior. More specifically, this study was designed to test the hypothesis that because CB may interfere with the processing of motion information, individuals with CB could place a higher weighting on more reliable alternative sources of information to guide the control of steering.

The finding that humans are quite good at estimating heading from optic flow in the presence of other moving objects^34–36^ suggests that the simple omission of visual information within a CB field could have a negligible impact on steering. We are equipped with neural mechanisms that facilitate this flow-parsing^37^, to the point where human heading direction judgments are biased only when large objects cross over the FoE^36^ up to between 1.9-3.4 degrees of error^36^. One reason why humans can detect heading direction so well in the presence of multiple moving objects is that flow from self-motion is spatially and temporally correlated^38^. This means that information from any one portion of the flow field is highly redundant with information from the rest of the flow field, and that the removal of any one portion may have minimal impact on the ability to perceive heading.

Alternatively, if CB does not manifest itself as an omission of visual information but instead as a source of noise in visual motion processing^39^, then there are several reasons to predict that steering would be affected. Human heading judgments can be biased by perturbations in the flow field^40^ in those without CB. Issen et al. (2015)^40^ showed that when the FoE was masked, judgments of heading involved the integration of optic flow across the visual field. Indeed, when an entire quadrant presented information inconsistent with the rest of the field, this biased the final estimate. Similarly, Layton and Fajen (2016)^34^ found that the FoE from forward self-motion can be biased by the local FoE’s of objects in the field of view. These findings suggest that judgments of heading may not be robust to variations across the flow field (but see^41^), and they raise the possibility that heading judgments may also be affected by elevated internal noise, such as is found in a cortically-damaged visual system^39^. The fact that individuals with CB display measurable biases in lane position when driving^9^ is consistent with this hypothesis. However, an alternative hypothesis proposed by Bowers (2010) is that CB participants may be leaving a margin of safety in the presence of obstacles that might unexpectedly emerge from their blind field.

To date, research into how CB affects optic flow processing has generated conflicting results. Some studies reported that CB patients remained sensitive to optic flow stimuli in their blind field^12,42^. Others found no differences in the behavior of individuals with CB when walking in the presence of optic flow subjected to either their visually-intact hemifield or cortically-blind hemifield, suggesting a potentially larger influence of extrastriate motion processing areas in CB patients^43^. A better understanding of how CB impacts steering, which has been found to rely heavily on the use of motion information, could help inform future vision rehabilitation approaches, and has the potential to increase safety of CB drivers and those around them.

When evaluating the effect of CB on motion processing for steering, it is also important to consider that some individuals exhibit compensatory eye movements while driving, presumably to monitor for potential obstacles such as pedestrians or other vehicles^10,44–48^, a behavior that can affect lane positioning. Compensatory eye movements may occur even in the absence of visible hazards. To consider how compensatory eye movements might influence steering, it is helpful to first understand where visually healthy individuals look when steering. In one study, Land and Lee observed that drivers fixated the tangent point when negotiating curves^49^. Subsequent research revealed behaviors that challenged this hypothesis^15^, including the presence of optokinetic nystagmus^16,50,51^. Generally, nystagmus is suppressed when a stable fixation point is present against a moving background^52,53^, but the fact that it is not suppressed when gaze is directed towards the tangent point, which is stable in the visual field, suggests that drivers may actually fixate the ground plane near the tangent point rather than the tangent point itself^51^. Such inconsistencies have prompted researchers to explore alternative explanations for why gaze tends to be directed toward the inside bend of a curve while steering. Some of the alternative explanations also highlight the role of eye movements in shaping the pattern of optic flow on the retina^15,54,55^, as well as the potential contribution of extraretinal sources of information about gaze direction^56,57^. Additional investigations could reveal if and how the presence of a blind field might disrupt coordinated gaze and steering behaviors.

To address whether CB affects the processing of optic flow in a manner that impacts steering behavior, this study compared the behavior of CB and age-matched controls in a virtual reality steering task in which the quality of optic flow was systematically manipulated. The participant’s task was to steer so that their head remains centered within the clearly demarcated single-late roadway. The manipulation of optic flow quality was accomplished indirectly, by varying feature/texture density in the virtual visual environment, an approach shown to be effective in Giguere et al. (2024)^58^, Warren et al. (1989)^59^, and Alefantis et al. (2022)^29^.

Prior studies provide insight that can be used to make task-specific predictions of the role of optic flow and information from visible road edges on the visual guidance of steering in our virtual reality task. Lane edges provide splay angle information, and equalizing left and right splay angles specifies a centered lane position in a way that is relatively insensitive to changes in vehicle orientation^60,61^. Li and Chen^27^ explored the relative influence of information from lane edges and optic flow, finding that, although observers primarily rely on information from the lane edges, lane-keeping performance nevertheless improves as the optic-flow field as optic flow density is increased, indicating that drivers can also exploit optic-flow–based self-motion information when it is available. In a subsequent study that used a virtual reality display very similar to the one used in the present study, this transition was presented as a tendency to increasingly hug the inner road edge as optic flow density increased  ^58^. Multiple investigations of steering towards a distant target in the absence of lane edges found that enriching optic flow (e.g., adding foreground motion or increasing flow density) improves steering precision and reduces response delay when steering towards a target in the absence of lane edges, in parallel with improved heading-perception accuracy^28,29^.

These findings motivated clear expectations for the control group in the present study: reducing optic flow density should reduce the influence of optic-flow–based heading information on steering, which we expected to manifest as less corner cutting and increased steering/lane-position variability relative to richer optic-flow conditions. Our central prediction was that these optic-flow–dependent changes would be attenuated in CB, because motion processing after V1 damage can be limited by elevated internal noise^39^, potentially reducing the reliability (and thus behavioral weighting) of optic flow for online steering control.

The use of virtual reality with integrated eye tracking allowed for the monitoring of eye and head movements to investigate whether CB participants attempted to compensate for their visual deficits through the use of compensatory gaze strategies while steering. In this study, which was absent of obstacles, we hypothesized that the presence of a blind field could cause shifts in gaze that were intended to bring more of the upcoming road edges into the intact visual field. Prior work suggests that this would have the unintended consequence of biasing the future path in the direction of gaze^15,21,26^, presumably in a manner constrained by the boundaries of the road. These possibilities motivated the need to record and analyze gaze data to promote a more complete comparison of behavioral differences between groups under different optic flow conditions.

## Methods

### Apparatus

Data were collected on two computers at separate locations. One computer was located at the Rochester Institute of Technology (RIT) and featured an AMD Ryzen 9 5950X 16-core processor, an NVIDIA GeForce RTX 3080 Ti graphics card, and 32.0 gigabytes of DDR4 RAM. A second computer was located at the University of Rochester (UR) and featured an 11th gen Intel(R) Core(TM) i7-11700K processor, an NVIDIA GeForce RTX 3080 graphics card, and 16.0 gigabytes of DDR4 RAM.

Both computers used the Windows 10 operating system. The experiment was created using Unity, version 2021.3.0f1^62^ (https://unity.com/releases/editor/whats-new/2021.3.0), and data analysis was done in a Python 3.9 virtual environment^63^ (https://www.python.org/downloads/release/python-390/) using Numpy version 1.26.3^64^ (https://numpy.org/devdocs/release/1.26.3-notes.html), Pandas version 2.1.4^65^ (10.5281/zenodo.3509134), and Matplotlib version 3.8.0^66^ (https://pypi.org/project/matplotlib/3.8.0/). Data collection and the randomization of trials and block order was completed with the help of the Unity Experiment Framework package for Unity (UXF^67^ (https://github.com/immersivecognition/unity-experiment-framework). Eye tracking data was recorded using Pupil Capture version 3.5.1 from Pupil Labs^68^ (https://pupil-labs.com/releases/core-v3-5).

Stimuli were delivered on an HTC Vive Pro headset^69^ (https://www.vive.com/) with a nominal binocular field of view of 107$^{\circ }$ along the horizontal and 108$^{\circ }$ along the vertical^70,71^. Depending on the position of the participant’s eyes with respect to the headset, this field of view could vary slightly. The HTC Vive Pro was fitted with a Pupil Labs eye tracker that recorded 400x400 pixels resolution eye images at 120Hz per eye. This was done for all but two participants, for whom eye images were recorded at 192x192 pixels on account of an experimenter error made at the time of data collection. The participants’ head position was continuously tracked with the integrated head tracking provided by Vive using two Lighthouse Base stations (version 1).

Participants used a Logitech G920 steering wheel^72^ (https://www.logitechg.com/en-us/products/driving/driving-force-racing-wheel.html) to navigate the simulation. The Logitech G920 offers a few benefits for customizing wheel response including the ability to fine-tune general wheel sensitivity and the ability to change the centering spring strength, which alters the strength with which the wheel returns to the centered position. In this study, we chose wheel sensitivity to be 40 and centering spring strength to be 40, since these parameters were reasonable enough to encourage quick adaptation to the experiment during practice.

### Task environment

**Figure 1:**
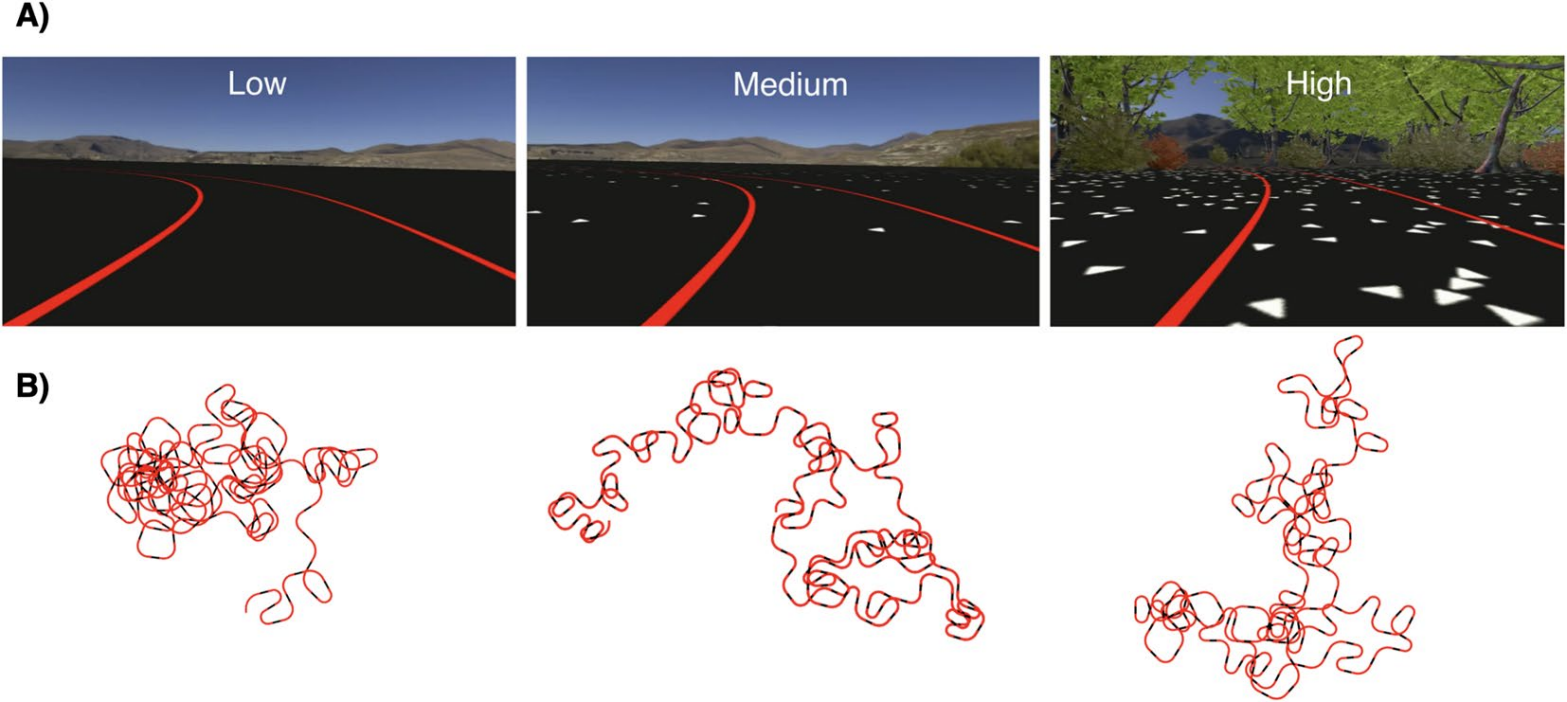
(**A**) A participant’s view of the task environment from inside the virtual reality headset. Participants were asked to stay centered within the two red lines. The three optic flow density conditions implemented in this study are shown, with increasing density from left to right. A screen recording of the task can be found in https://youtu.be/_3Mginft_zk. Due to the reduced resolution and video compression, some textures (e.g. distant road edges) may appear at a lower contrast than in the true simulation. (**B**) Top down views of the procedurally-generated roadway from two participants as they steered in VR. The straight parts of each trial are indicated in black while the curves are displayed in red. Each curve has a radius of curvature 35m, 55m, or 75m. Turns were removed from the scene once traveled to preserve the sensation of a continuous, never-ending roadway. The addition and removal of turns was seamless and imperceptible to the observer.

The task environment was designed to explore the relationship between optic flow and steering behavior, which should be considered a subtask of the superordinate task of driving. Participants were immersed in a virtual reality environment in which a road, outlined by thick, high-contrast red lines, wound its way over a flat, black surface (Fig. 1A). The car dashboard and steering wheel were removed from the visual field to increase the angular extent of the visual stimulus and the surrounding flow field. The environment was kept visually simple with the intention of reducing alternative strategies for visually guided control that did not rely on the use of optic flow. Although the solid-lined road edges provided an additional source of visual information about self-motion in the environment, their primary purpose was to define the path to be ”driven” in order to complete the task.

In anticipation of the possibility that some older observers would be unable to wear corrective lenses inside the head mounted display, choices in the color and appearance of the textured ground plane, roadway, and foliage were made with the intention of creating high contrast optic flow information visible even under the influence of blur. Similarly, the color of the bright red road edges was chosen to ensure that it was of sufficient contrast relative to the ground plane for the purpose of increasing path visibility far into the distance.

#### Road design

The road was composed by alternating individual turn segments with straight segments (Fig. 1B). As part of the trial randomization process, turns were appended to the road in real-time, with two turns ahead present at all times. These upcoming turns were positioned far enough ahead to blend seamlessly into the environment, giving the impression of a never-ending roadway. Once the participant navigated past a turn, it was promptly removed from the scene while out of view. Verbal feedback from participants confirmed that this on-the-fly addition and removal of road segments occurred without their awareness, creating the illusion of a continuous path. This methodology facilitated the ability to present each participant with the same pool of trials/turns in a randomized order, thus reducing the potential influence of both practice and fatigue on performance.

#### Manipulation of turn direction and turn radius

Trials were equally split between left and right turns. Each road bend was 100 meters in length, with 20m of straight road on either end of the curve. Trials were also equally divided into three turn radii: 35, 55, or 75m. Road curvature was varied deliberately to reduce the likelihood that later turns could be navigated using motor responses learned on earlier trials without the use of optic flow.

#### Manipulation of optic flow density

The manipulation of optic flow density was carefully designed to support the observation of the main effect of interest; optic flow density on steering. The density was altered along an ordinal scale by adjusting the amount of objects and features in the scene that would contribute to flow from self-motion. This was done by changing the density of white triangles on the black ground plane as well as by adding or removing trees from view. These elements provided information about speed and heading direction, and the mountains present in the distance supplied information about rotation rate when turning. Since the mountains were always kept at a fixed distance away from the participant in the virtual environment, they yielded no information about translational flow from self-motion.

On the low flow-density trials, only the red road edges and the distant mountains were visible, rendered as a custom skybox (Fig. 1). On medium flow-density trials, white triangles were added to the black ground plane with minimal density (5 triangles per square meter). High flow-density trials featured an increase in triangle density to 53 triangles per square meter and the addition of trees and bushes on both sides of the road. These changes in flow density took place between turns/trials – in other words, changes occurred on the straight parts of the path that connected two consecutive trials.

### Experimental procedure and design

Each participant was instructed to ”steer naturally, while centering their head and body in the middle of the single-lane road”. This instruction promoted the ability to measure participant lane position from the center of the road or the inside road edge. Participants were also told to pause the experiment and/or take the headset off if they felt VR sickness.

Prior to being immersed in VR, participants were shown an example video depicting the view inside the headset while a person completed the eye tracking calibration procedure and the first few turns of the driving task. For the eye tracking calibration, targets appeared in a predictable pattern - the center target was presented before the right-most target, and subsequent targets followed a counter-clockwise pattern around a circle of constant radius. Prior exposure to this predictable pattern through the example video was critical for helping CB participants anticipate when a calibration target would be presented in their blind field and for helping them saccade in that direction when appropriate.

After the task had been described to all subjects, each person was asked to sign the consent form in order to participate. General information about their visual prescriptions, prior driving experience, and demographics were filled out as part of the form-signing process. Upon signing, each person was fitted with the Vive Pro and immersed in the virtual environment with the opportunity to practice steering along multiple turns until they felt comfortable. This usually lasted for about ten turns, at which point they had sufficiently adjusted to the steering sensitivity and visual changes between trials. During these practice trials, participants were also instructed on how to pause themselves (and restart) mid-experiment, should they need to rest due to VR sickness. They were told that they could also remove the headset if need be, in which case the eye tracking calibration would be repeated before restarting.

Before officially beginning experimentation, each participant was asked whether they wanted to remove the headset and take a break. Once ready, the experiment began with the eye tracking calibration sequence. Participants started their own motion along the virtual road whenever they were ready after completing the initial eye-tracking calibration tests monitored by the researcher.

Each participant was tasked with completing a total of 144 turns while moving at a constant speed of 19.0 m/s. Of these 144 trials, each trial type was repeated eight times, and the trials types were defined by combinations of the following parameters: optic flow density (low, medium, high), turn direction (left, right), and turn radii (35, 55, and 75 meters).

### Participants

Participants were divided into three groups: those with visual deficits on the left side of their visual field (left CB), those with deficits on the right side (right CB), and a group of controls. The ages of control group participants ranged from 49 to 68 (Mean: 57.4, SD: 5.6), of those with right-sided CB field deficits ranged from 40 to 69 (Mean: 54.0, SD: 8.7), and of those with left-sided CB field deficits ranged from 36 to 76 (Mean: 55.1, SD: 10.6). An independent-samples *t*-test comparing the control group to the two CB groups revealed no statistically significant differences between means (Control vs. Right CB: $t = 0.98$, $p = 0.34$, Control vs. Left CB: $t = 0.61$, $p = 0.55$). An *a priori* power analysis was conducted on mean lane position using GPower 3.1^73^ for a mixed-design ANOVA testing the Group $\times$ Optic Flow Density (within–between) interaction with three groups and three measurement occasions. The test assumed an effect size of 0.88 that was estimated using three members of each group, a Type I error rate $\alpha =.05$, and a nonsphericity correction $\varepsilon = 1$. The analysis indicated that a total sample size of $N = 15$ participants (i.e., 5 per group in a balanced design) would be required to achieve 80% power. Although a total of 38 participants enrolled in the study, four controls and three CB subjects were unable to complete the task due to VR sickness. One CB participant was excluded from all analyses for excessive differences in steering compared to all others which we interpreted as a failure to understand the task. The number of participants per group that contributed to the final dataset of steering behavior included 9 visually-intact older adults (8 male, 1 female), 11 left CB (10 male, 1 female), and 10 right CB participants (6 male, 4 female) for a total of 30 participants in the steering analysis. Although group sizes were slightly unequal, this degree of imbalance is minimal, and the total sample size substantially exceeds the number required by the power analysis; thus the sample size exceeds that required under these assumptions.

Note that, due to technical issues, a smaller number of participants’ gaze behavior was successfully recorded during task performance. The subset of participants per group that contributed to the dataset with *both* steering and gaze behavior included 5 controls, 4 left CB, and 7 right CB subjects.

Each cortically blind participant was thoroughly screened at the University of Rochester to ensure that they had no cognitive or motor impairments. The location of their blind fields were meticulously mapped using Humphrey perimetry (SITA Standard, 24-2 and 10-2 patterns, Goldmann size III stimuli). Monocular Humphrey visual fields were interpolated and averaged between 24-2 and 10-2 tests, as well as between eyes, to generate composite maps. We asked, but did not require, that our subjects wear contact lenses to improve performance and reduce VR sickness. Glasses were not worn in the headset to prevent scratching the VR lenses, and degradation of the eye tracking signal. Of the enrolled control participants, two drove normally (i.e., in everyday life) without correction, two wore contact lenses, and nine did not own contact lenses and wore glasses instead. Of the eleven participants with corrected vision, ten provided their prescription information when asked, of which six were near-sighted (average left/right eye correction was -5.20D/-5.08D) and four were far-sighted (average left/right eye correction was +3.5D/+3.74D). Of the cortically blind participants, eight had correction, with two wearing contacts and 6 wearing glasses. Six were near-sighted, with average left/right eye correction of -2.9D/-2.7D, while two were far-sighted with corrections of +3D/+3D and +4D/+4D, respectively. The remainder were uncorrected. Four t-tests confirmed that there were no statistically significant differences between the left-sided CB and right-sided CB groups in age, time since stroke, sparing of vision within the central $10^\circ$, or sparing within the central $24^\circ$ of the visual field. Table 1 displays each of the visual field maps for all CB participants. The noticeable variation in the location of spared peripheral fields will be revisited during the analysis of steering behavior.

**Table 1:**
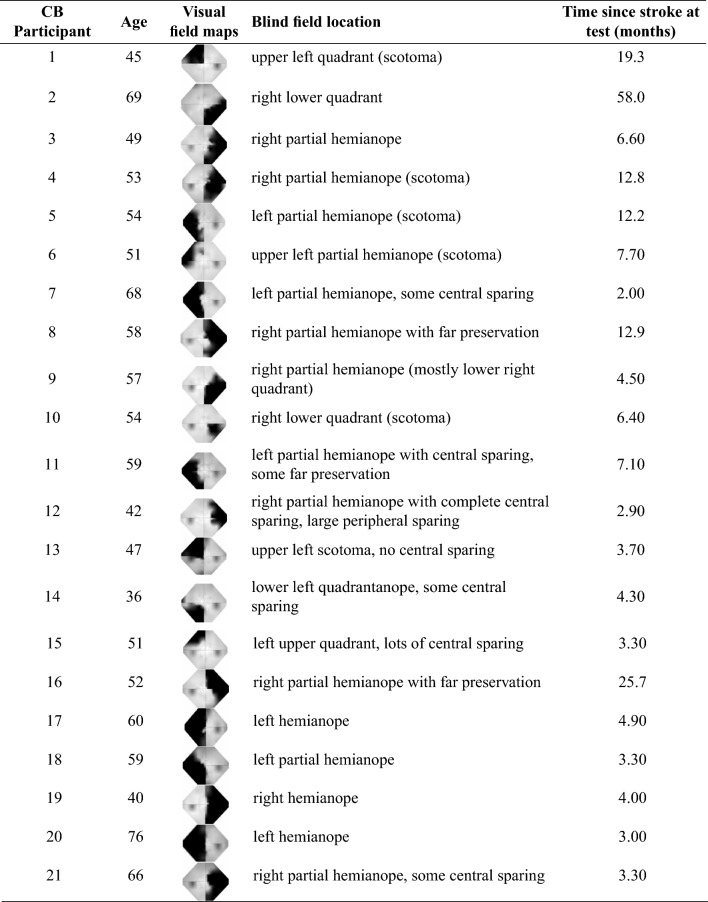
A summary of characteristics of the CB participants that contributed to analysis.

Each visual field map spans $27^\circ$ horizontally and $21^\circ$ vertically. The black shaded areas represent areas of visual impairment, while light gray areas represent intact vision. There are no significant differences between groups in sparing of the visual field within the central $10^\circ$ or central $24^\circ$.

All control participants signed consent forms approved by RIT’s institutional review board and were compensated $10 for their time. All CB participants signed a consent form approved by UR’s institutional review board and were compensated $15. All data collection at both locations was performed in accordance with the guidelines and regulations defined in the IRB-approved consent forms.

### Steering analysis

In order to analyze steering behavior, we measured the distance from the participant’s head to the inner road edge at every time step (sampled at Unity frame rate of 90 Hz). Since participants often cut corners when navigating the curves despite being instructed to center themselves in the lane, we chose to measure distance from inner road edge because this single metric could describe steering on both left and right turns. Each turn was constructed of a discrete number of vertices along the road center (more vertices for sharper turns), and the distance between the participant’s head and closest road vertex was measured at each time step. Offsetting these values by the half-width of the road ultimately yielded the distance from inner road edge at each time stamp. By recording these data for each trial type, the average trajectories across each turn radius and optic flow density were calculated by participant and across participants. Both the timestamps and steering data were interpolated over a fixed range for all participants, since the samples were not collected at equal time intervals. The interpolation was done using the ”interp” function from numpy (Python) at 90 Hz. Additional analysis of the steering data required averaging the time series distances from inner road edges along various segments of the road over time. Other dependent variables such as wheel angle and head orientation were recorded at Unity’s frame rate of 90Hz, and all custom road parameters were logged for trial-by-trial plotting purposes.

### Gaze analysis

We leveraged the Pupil Labs software for the estimation of gaze direction. This software supports uploading the raw eye recordings from which offline calibration can be implemented and gaze direction within the head-centered frame of reference can be estimated. Although the Pupil Labs’ framework provides an algorithm for segmentation of the pupil within the eye image using image processing, previous research has shown that trained computer vision algorithms can improve the 2D pupil detection prior to 3D gaze estimation with potential improvements to the accuracy, precision, and robustness of the estimate^74^. In an effort to improve gaze accuracy, data from each subject was passed through the EllSeg algorithm developed by Kothari et al. (2021)^74^ to produce offline detected pupils. The offline pupils were then used by the Pupil Labs software as part of the 3D gaze estimation process, which involved fitting a 3D eye model to the ellipse fits of the identified pupil within each eye image. Although the default behavior of Pupil Labs software involves the continuous updating of its 3D model of the eye, we found results were much more accurate when the eye models were fit to hand-selected durations of each recording. These sections were selected by the experimenter to prioritize a small number of blinks and a range of eye-in-head orientations. Selections were used to estimate gaze, and visual inspection of the calibration sequences at the beginning and/or end of the experimental session were used for qualitative assessment of the model fitting process. Following a positive qualitative assessment, gaze estimation was then performed across the rest of the video using the frozen fitted eye model prior to quantitative evaluation of the gaze estimation results, using the sequence at the end of the experimental session. For four participants, this sequence was missing due to participant VR sickness or experimenter error, in which case the initial calibration sequence was utilized. Overall, the average accuracy and precision results of gaze estimation were as follows: age-matched controls (n=5), accuracy=2.34$^{\circ }$ and precision=0.10$^{\circ }$; CB (n=16), accuracy=2.76$^{\circ }$ and precision=0.15$^{\circ }$. Samples below the confidence level of 75% set by the Ellseg-gen algorithm^75^ were replaced with null entries. Low-confidence samples were often the result of temporary issues related to illumination or occlusion. The final estimates of eye orientation within the head were exported to text file (*.csv) for subsequent analysis in Python.

Each row in the exported gaze positions contained data from each eye camera operating asynchronously at 120Hz, for an effective sampling rate of 240Hz. During pre-processing, the head orientation data was up-sampled to the same 240Hz frequency using interpolation based on the timestamps provided by Pupil Labs. The monocular data were merged into a single cyclopean gaze estimate using numpy’s nanmean() function to average the two eye vectors, accounting for potential null entries from low confidence in one eye or both. The estimated gaze vectors were normalized. Each cyclopean estimate of eye orientation within the head ($\overrightarrow{EIH}$) was then transformed into a gaze-in-world (GIW) direction vector  ($\overrightarrow{GIW}$) using the head’s transformation matrix. To eliminate translation effects during this calculation, the translation components of the matrix were set to zero.

**Figure 2:**
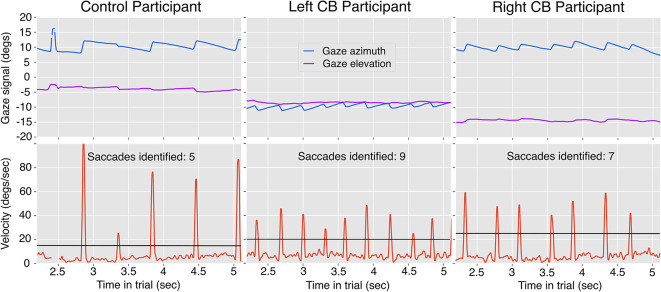
Representative gaze behavior from three single trials of three different participants. Only the middle 40% of each trial is shown because all statistics are calculated over this portion, and variations in the signal are more easily visualized with an expanded X axis. The top row includes the gaze azimuth and elevation position time-series signals, and the bottom row displays the corresponding velocity signals post-filtering $\sqrt{\dot{az}^2 + \dot{el}^2}$. All examples are turns with radius 75m which had smaller magnitudes of gaze azimuth, and thus were preferred for ease of visualization. Each column represents one participant from each of the three groups. The number of saccades identified by the custom saccade algorithm is indicated in the text. The black horizontal lines mark the saccade speed threshold used for each participant.

From these gaze-in-world gaze estimates, azimuth and elevation were calculated relative to the body’s forward position and flat ground plane orientation, which were sampled at the start of the experiment. The “body forward” direction was identified when participants held the steering wheel and were instructed to look “straight ahead” As shown in Fig. 7, the distribution of gaze azimuth positions was bimodal, given that participants looked in the direction they were turning. For this reason, statistical analyses of gaze azimuth magnitude were run on the absolute value of gaze azimuth. Visual inspection of these velocity signals revealed a pattern of nystagmus present in all optic flow density conditions, carried primarily by variation in the azimuthal direction, as presented in the top row of gaze signal in Fig. 2. In contrast, the gaze elevation signal remained relatively constant. The relatively constant gaze elevation was also confirmed by revisiting the recorded videos of each participant’s view from inside the headset with overlaid gaze position. Because predictions concerned the possible covariation of gaze direction with changes in visual conditions while steering, the estimate of gaze elevation and the oscillating gaze azimuth signal were averaged across the middle 40% of each turn’s length to produce a single gaze azimuth and elevation value for each trial. Similar patterns of small-amplitude optokinetic nystagmus have been observed in previous studies when steering around corners under simulated driving conditions^50^, real-world driving scenarios,^16,51^, and when steering bicycles^76^.

Analyses also investigated differences in saccade frequency across group and visual condition. Saccade identification was performed upon the gaze velocity signal, which was computed at each time step from the azimuth and elevation position and associated time stamps. Velocity along azimuth and elevation was then filtered using subsequent rolling mean and median filters with kernel sizes of five (a nominal duration of 20 ms assuming a constant sampling rate of 240Hz). The filtered azimuthal and elevation velocities were combined into a single, directionless velocity signal: $\dot{\gamma } = \sqrt{\dot{az}^2 + \dot{el}^2}$. Saccades were identified from this signal using a threshold that was allowed to vary per participant on the basis of the noise-floor of the gaze velocity signal (see Fig. 2 for example thresholds denoted by the blue horizontal lines). The thresholds averaged around $25^\circ$ per second and ranged from $15^\circ - 60^\circ$ per second. The noise-floor of the two participants for which the threshold was set to 60 was higher due to manual error in setting the eye tracking parameters at the time of data collection. This led to a pixel resolution of 192x192 for the eye images rather than 400x400. Although saccades exist at a wide range of spatial scales and amplitudes^77^, we found that these thresholds were suitable for counting the number of saccades that resulted in the repositioning of the fovea to a new area of the visual scene.

The number of saccades was determined by the number of segments with consecutive indices that exceeded both the velocity threshold and the minimum saccade duration of 25 ms. Outlier trials were removed if the number of identified saccades within a trial exceeded the range of three standard deviations from the mean by participant.

### Statistical analysis

Statistical analyses were implemented in Jasp version 0.19.3^78^. Power analyses were conducted using GPower version 3.9.1.7^73^. Mixed-design ANOVAs were applied to each dependent variable, including distance to inner road edge along the middle 40% of the road, maximum departure from the center lane, lane position variability, gaze azimuth along the middle 40%, gaze elevation along the middle 40%. Each ANOVA included the following independent variables: turn direction, turn radius, optic flow density, and subject group. Preliminary tests were applied to ensure equality of variances and if sphericity was violated, the violation was corrected using the Greenhouse-Geisser method. Significance tests were evaluated at an alpha of 0.05. All p-values from post-hoc analyses were interpreted following Bonferroni adjustment.

Additional t-test analyses were performed to assess differences between left-sided CB and right-sided CB participant groups. Differences are reported in the participant section above. Assumptions of normality and equality of variance were checked for each t-test and corrected where necessary using log transformations to address data normality and Welch’s t-test for violations of variance equality.

## Results

### Steering behavior

**Figure 3:**
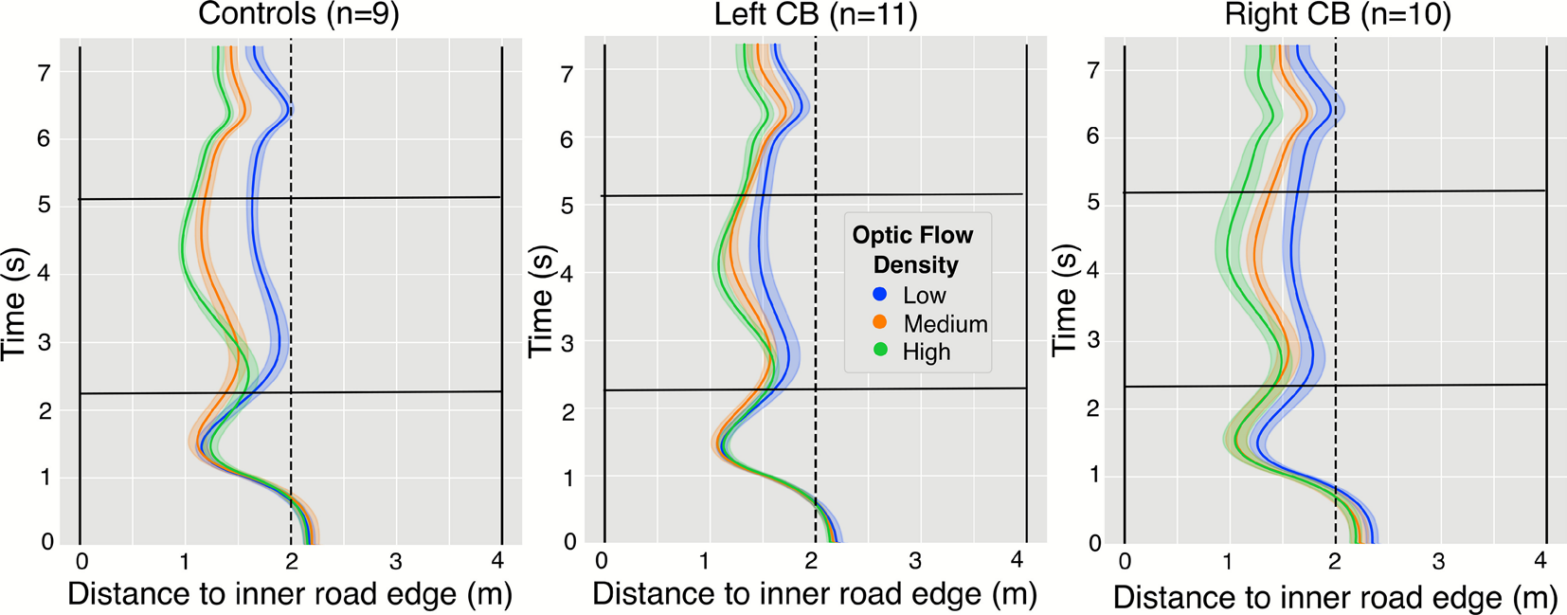
Average lane position over time for all three participant groups across trials with turn radius 35m, which was the tightest of the three turn radii. Lane position is measured from the inner road edge. Values closer to zero on the horizontal axis indicate average positions closer to the inner edge of the road, regardless of the turn direction. The 95% confidence intervals are depicted as shaded areas centered around the mean. Each trial begins at t=0 and ends at t=7 on the y axis. The curved portion of the road falls within approximately t = 1 to t = 6 s, while straight parts occur around t=0 to t=1, and t=6 to t=7. The edges of the 4 meter-wide, single-lane roadway are indicated by the vertical black lines, while the center of the road is dotted. The black horizontal lines indicate the beginning and end of the central 40% of the turns.

**Figure 4:**
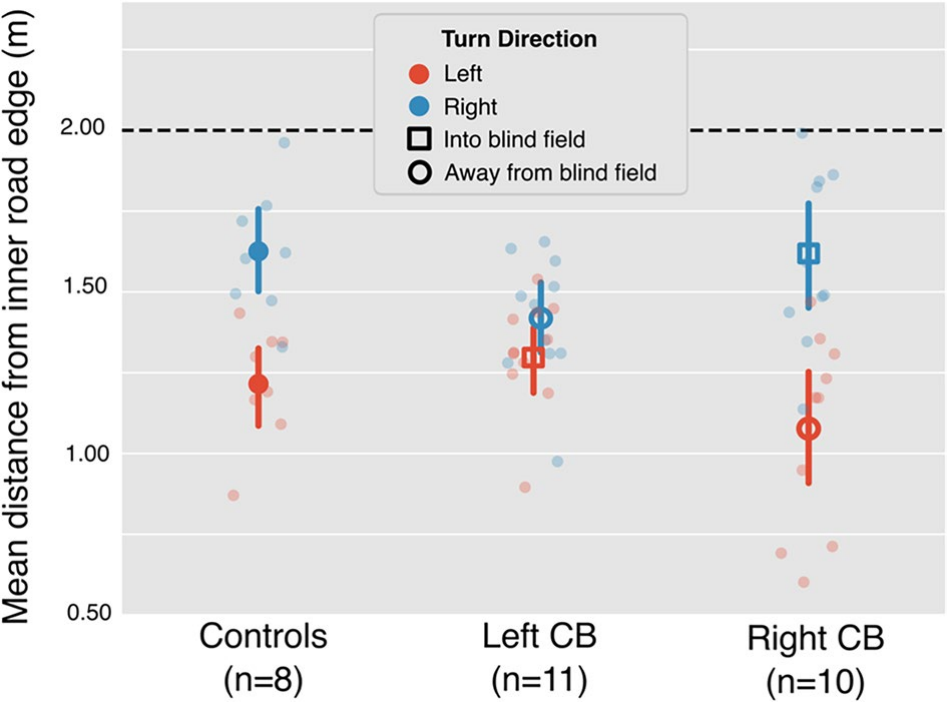
Average lane position from the inner road edge while steering on the middle 40% of each turn. Data is separated by turn direction and participant group to illustrate differences in average lane biases. Individual points (semi-transparent) represent data from each participant within each group. Error bars on the large, dark symbols represent the 95% confidence intervals across participants. One participant from the control group was removed from turn direction analysis due to opposite corner cutting behavior from learning to drive on the left side of the road. Data points of turns into the blind fields are represented by empty circles, and square data points indicate turns away from the blind field.

Figure 3 presents the average paths of participants as they approached and traversed 35m radius bends in the road. Each trial began with a segment of straight road 20m in length (roughly from 0-1 seconds), followed by a 100m turn of constant curvature (from 1-6 seconds), complete with another 20m straight road portion (from 6 to 7 s). The average trajectories are broken down by subject group and flow density. These trajectories show several aspects of steering behavior common to all participants. All participants tended to be slightly biased towards the outer edge of the road when approaching the turn, at time t$=$0. They crossed the road’s center at approximately 0.8 s into their traversal, about 0.2 seconds before entering the curved portion of the road.

Participants negotiated the central part of the bend during the middle 40% of each trial, which is indicated by the horizontal black lines at $\approx 2.2$ and $\approx 5.1$ seconds. This segment is also where differences in steering behavior related to optic flow density first became evident, as is indicated by visual separation of the three colored trajectories within each panel of Fig. 3. For this reason, subsequent analysis of steering and gaze behavior involve averaging across this duration.

All participants cut corners more on left turns than right turns (F(1,26) = 42.50, $p<0.001$, $\eta _p^2 = 0.620$, Fig. 4A), and the significant interaction of turn direction with group suggests that this effect was modulated by the presence of cortical blindness (F(2,26) = 6.15, p=0.007, $\eta _p^2 = 0.321$). Post hoc analysis revealed that significant differences in turn direction lie between left and right turns for the control group ($p=0.006$) and the right-sided CB group ($p<0.001$), but not the left-sided CB group. While visually, it seems possible that the left-sided CB group was biased away from their blind field when steering, the differences in lane positions of left-sided CB participants and controls within a given turn direction was insignificant.

**Figure 5:**
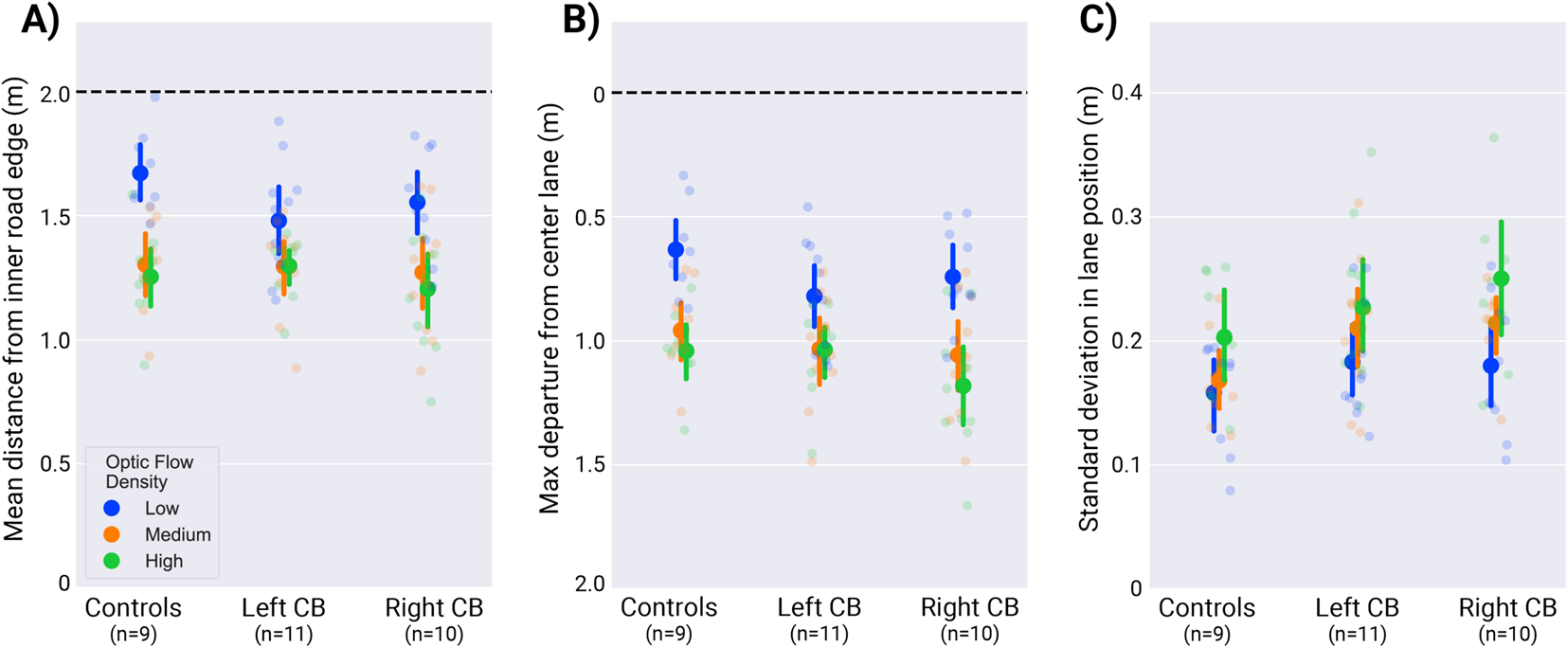
Steering related data broken down by group and optic flow density. Data is averaged over the middle 40% of each trial and separated by group. Individual points represent participant-level data. Error bars display the 95% confidence intervals across participants. (**A**) Mean lane position from the inner road edge separated by optic flow density. (**B**) Maximum departure from the center lane. (**C**) Standard deviation in lane position.

**Figure 6:**
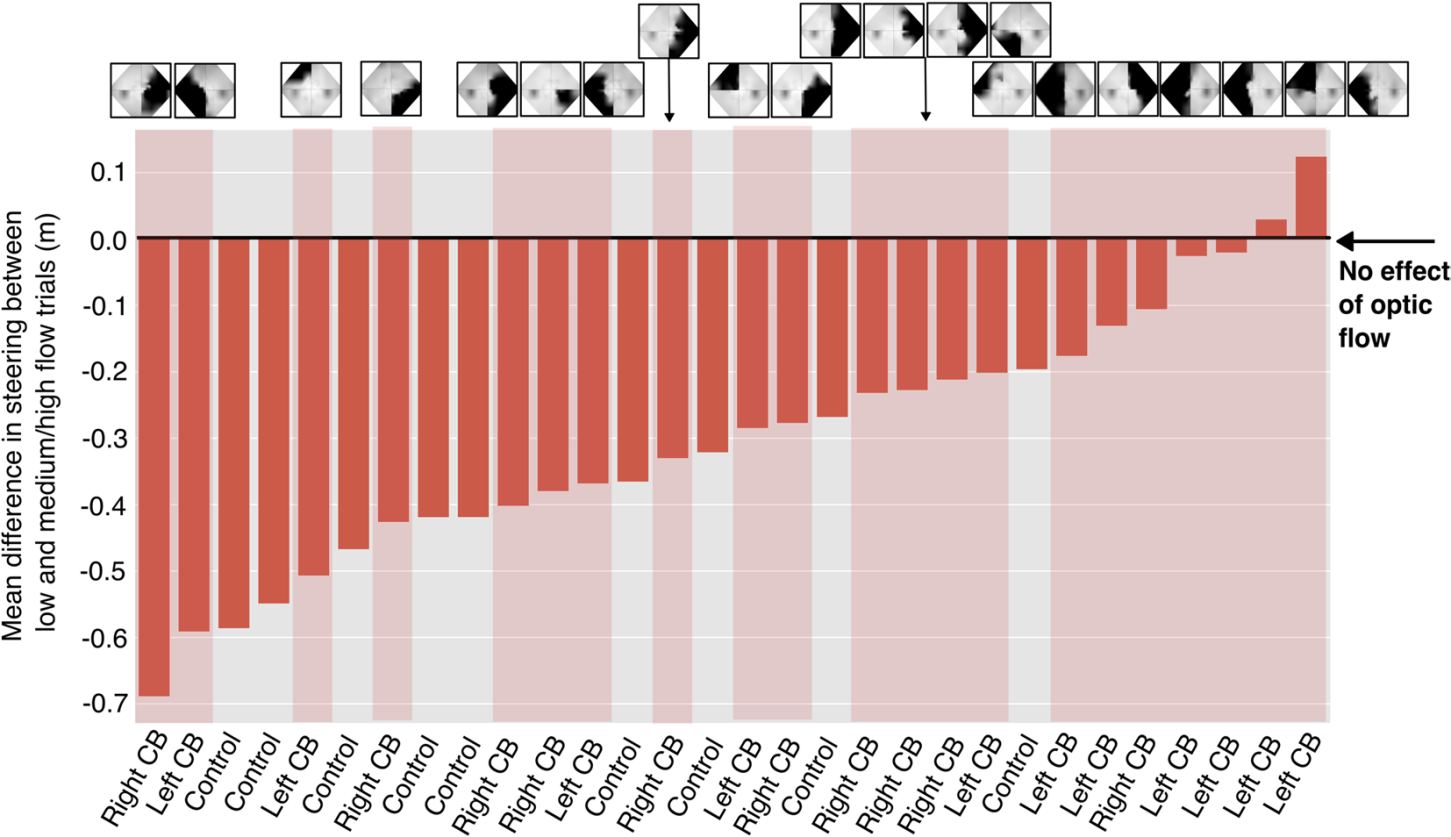
Differences in lane positions between low flow density trials and medium/high flow density trials for each participant. Smaller bars, or those closer to zero, represent smaller effects of optic flow density on steering. Participants are increasingly ordered according to their mean differences, and CB participant data are indicated in shaded red. Each CB subject’s visual field map is located above their column of data, and the black areas within denote their visual deficit. The figure illustrates how CB participants are generally more concentrated to the right of the bar graph, highlighting their minimized response to steering between low and medium/high density trial conditions. There is no clear relationship between area of visual deficit and steering behavior. This figure also emphasizes individual variability in steering behavior.

The effect of optic flow density on mean lane position is presented in Fig. 5A. There was a significant effect of optic flow on lane position. Participants cut corners less in the low flow-density condition, in which there was rotational flow from distant landmarks but no optic flow arising from forward translation $(F(1.42, 38.42) = 71.97$, $p<0.001$, $\eta _p^2 = 0.727$). Post-hoc tests suggested that the differences were between low/med: ($p<0.001$), and low/high ($p<0.001$) conditions, but not (med/high: $p=0.597$). The central hypothesis concerned the interaction with subject group, which was significant: F(2.85, 38.42) = 2.92, $p=0.049$, with a large effect size ($\eta _p^2 = 0.178$). Despite the significant interaction of group and optic flow at the omnibus level, pairwise comparisons across groups in Fig. 5 with matching optic flow density (e.g., across the three blue data points) failed to reach significance. Instead, the interaction reflects how differences in how optic flow levels modulate max divergence within groups.

To more fully capture the effects of our empirical manipulations on steering behavior, we performed additional analyses examining maximum departure from the center lane and lane position variability. Figure 5B presents maximum departure from the center lane for each group and optic flow condition. Just as with mean lane position, the ANOVA reported the interaction of optic flow density and subject group to be significant ($F(3.22,43.46)=3.87$, $p=0.014$, $\eta _p^2=0.223$), but the subsequent post-hoc comparisons showed no reliable between-group differences within any single optic-flow level. These differences were once again carried at the within group level. For all groups, maximum departure was reliably lower on low density flow trials than on medium and high (low–medium, all $p<0.003$; low–high all $p<0.01$). Medium and high did not reliably differ for controls/left-CB, whereas right-CB showed a significant medium–high difference ($p=0.045$). Note that this result differs from that of mean lane position, for which the Left CB group did not demonstrate differences between optic flow levels. This analysis suggests that all groups were affected by the optic flow manipulation and that, when proximal/foreground flow was available in the medium or high flow conditions, it led to lower values of maximum departure. The analysis also revealed main effects of turn direction, turn radius, and optic flow density on max departure (turn direction: $F(1,27)=23.18$, $p<0.001$, $\eta _p^2=0.462$; turn radius: $F(1.37,36.88)=9.73$, $p=0.001$, $\eta _p^2=0.265$; optic flow density: $F(1.61,43.46)=100.71$, $p<0.001$, $\eta _p^2=0.789$). There was also a strong turn radius $\times$ optic flow density interaction ($F(3.79,102.19)=25.55$, $p<0.001$, $\eta _p^2=0.486$), indicating that the magnitude of the optic-flow effect was more pronounced at greater road curvatures. Max departure did not differ reliably across groups overall (subject group: $F(2,27)=1.05$, $p=0.365$, $\eta _p^2=0.072$), and other interactions involving group were not significant (e.g., turn direction $\times$ group: $F(2,27)=2.98$, $p=0.068$, $\eta _p^2=0.181$; turn radius $\times$ group: $F(2.73,36.88)=0.71$, $p=0.539$, $\eta _p^2=0.050$).

A measure of standard deviation/variability in lane position is presented in Fig. 5C. An ANOVA revealed no evidence that lane-position variability differed by subject group overall ($F(2,27)=1.84$, $p=0.178$, $\eta _p^2=0.120$), and interactions involving subject group were not significant, including optic flow density $\times$ subject group and turn radius $\times$ optic flow density $\times$ subject group (optic flow density $\times$ subject group: $F(3.944,53.248)=1.06$, $p=0.383$, $\eta _p^2=0.073$; turn radius $\times$ optic flow density $\times$ subject group: $F(5.150,69.520)=0.53$, $p=0.759$, $\eta _p^2=0.038$). However, there were strong main effects of turn radius and optic flow density, as well as a significant turn radius $\times$ optic flow density interaction (turn radius: $F(1.269,34.251)=155.06$, $p<0.001$, $\eta _p^2=0.852$; optic flow density: $F(1.972,53.248)=24.76$, $p<0.001$, $\eta _p^2=0.478$; turn radius $\times$ optic flow density: $F(2.575,69.520)=22.52$, $p<0.001$, $\eta _p^2=0.455$), indicating that the effect of optic flow on lane-position variability depended on road curvature. Turn direction did not show a reliable main effect ($F(1,27)=0.93$, $p=0.343$, $\eta _p^2=0.033$).

### Was the interaction of optic flow and group carried by a few individuals?

Because the visual deficits of CB patients varied in nature, additional steps were taken to better understand whether the significant interaction of subject group and flow density on lane position seen in Fig. 5 was characteristic of all members of the CB group, or whether the effect was driven by a few individuals. To illustrate the variety of steering behavior in our participants, Fig. 6 presents the *differences* in mean lane position between optic flow density conditions for each individual ($\Delta \rho =0.5\times (\rho _{l}-\rho _{m} + \rho _{l}-\rho _{h})$, where $\rho$ is lane position relative to the inside road edge, and the various levels of optic flow density are denoted as *l*, *m*, and *h* for low, medium, and high, respectively). It is notable that CB participants tended to have smaller differences in steering as a function of optic flow density which is also evident by the higher concentration of shaded red columns in Fig. 6 towards the right side of the X axis. Despite the increased concentration of red columns to the right of Fig. 6, a Kruskal-Wallis test on the ranked data between groups was insignificant. While this figure does not reveal outlier CB participants that could be responsible for the significant effect of flow density and subject group, it does emphasize individual variability in steering as a function of optic flow in our task and implies that some CB participants were less sensitive to optic flow density when steering in this task.

A closer look at the order of visual field deficits in Fig. 6 prompted a follow-up correlation analysis to investigate whether the location of visual deficits in CB patients in the upper vs. lower hemifields, or the left vs. right hemifields, would predict the magnitude of steering differences between optic flow conditions. We ran four correlations (Pearson’s) using a measure of the visual deficit area for each patient (squared degrees) compared with the mean differences in steering as displayed in Fig. 6. For those with a left-sided visual deficit, the steering differences between optic flow density conditions were smaller and correlated to their deficit area (moderate correlation, r = 0.412). The opposite was true for those with right-sided deficits, where greater visual deficit areas tended to yield greater differences in steering between flow conditions (weak correlation, r = -0.283). There was also a moderate correlation of steering with deficit area in the upper vs. lower visual fields across all CB subjects (r=0.443 for upper, r=-0.384 for lower).

Although the main focus of the present study was not about the effect of turn radius on steering behavior, additional effects of turn radius and turn radius with optic flow density were found on average lane position turn radius: $F(1.40, 37.92) = 7.84$, $p = 0.004$, $\eta _p^2 = 0.225$, turn radius * optic flow: $F(3.20, 86.27) = 6.98$, $p<0.001$, $\eta _p^2 = 0.205$). Importantly, these effects have insignificant interactions with subject group, suggesting that patients are steering similarly to controls with respect to turn radius.

**Figure 7:**
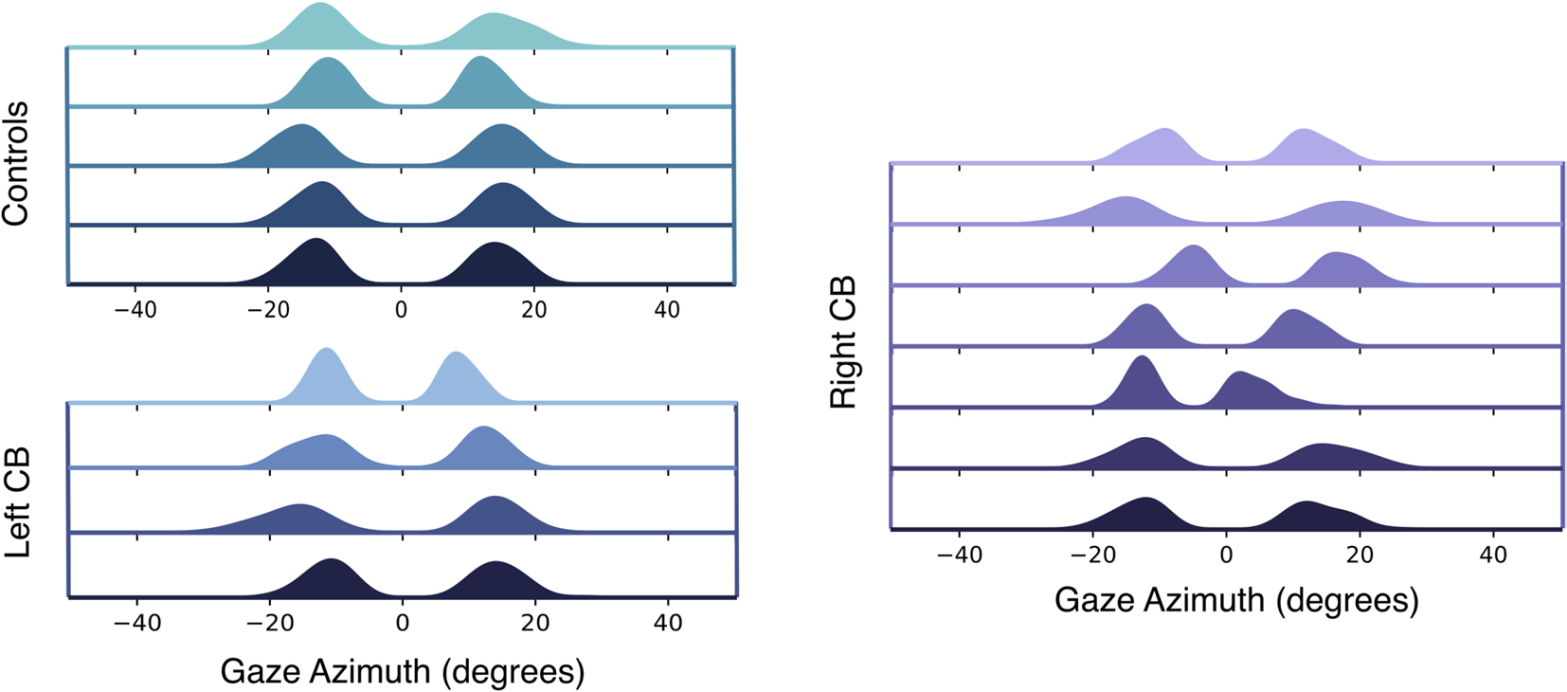
Distribution of within-trial gaze azimuth positions for the subset of participants that provided usable gaze data (see Methods for details). Each row represents data from one participant. Zero degrees represents the recorded direction the body faced in the seat upon session start. The bimodal distribution is present as a result of fixations to the left and right of the road on left and right turns respectively. Generally, gaze is symmetrical about zero for all participants, with the exception of a couple of participants in the right-sided CB group. If CB subjects exhibited compensatory scanning towards their blind fields, gaze distribution would be shifted left for left-sided CB, and right for right-sided CB.

### Does pooling left and right CB into a single group offer a more sensitive test of the effect of optic-flow?

In the steering analyses reported above, repeated-measures ANOVAs of mean and maximum divergence from the inner road edge yielded a significant group $\times$ optic flow density interaction, whereas Bonferroni-adjusted post-hoc comparisons revealed no reliable between-group differences at any single optic-flow level. This pattern indicates that the interaction reflects differences in the *within-group* modulation of divergence across optic-flow conditions rather than clear separation between groups at specific flow densities. Accordingly, the omnibus interaction should be interpreted conservatively and does not, by itself, constitute strong evidence that CB participants are differentially affected by optic flow relative to controls.

We assessed whether this conclusion depends on how CB participants are grouped, and hereby demonstrate that the interpretation remains unchanged when all CB participants were treated as a single group (controls vs. CB). This additional “pooled” analysis was motivated by the consideration that subdividing CB participants into left- and right-sided groups reduces the per-group sample size, potentially limiting sensitivity to effects that are small or not consistently expressed across individuals. Although the *a-priori* power analysis reported in the Methods section suggested adequate power to detect a Group $\times$ Optic Flow interaction of the magnitude observed in pilot data, the pilot-based effect-size estimate used for that calculation is variable at smaller sample sizes. At the same time, collapsing across hemifield is not unambiguously power-increasing because turn direction interacts with the side of the blind field: left and right turns elicit systematically different steering behavior even in visually intact drivers, and for CB participants the direction of the turn can be toward or away from the blind field depending on the side of the deficit (see Fig. 4). Pooling left- and right-sided CB therefore mixes trials in which the same nominal turn (e.g., a left turn) has different functional relationships to the blind field, increasing within-group variance and potentially attenuating any true group differences in optic-flow modulation. For this reason, the pooled analysis is reported as a complementary robustness check rather than as a replacement for the primary three-group approach.

An *a-priori* power analysis conducted for a mixed-design ANOVA testing the interaction with 2 groups assuming an effect size of $f = 0.74$, $\alpha = 0.05$, and nonsphericity correction $\epsilon = 1$, indicated that a total sample size of $N = 14$ participants (7 per group) would provide 80% power (actual power = 0.87) to detect the interaction. This indicates that the group size of 9 controls and 21 CB participants is sufficiently powered to test hypotheses related to the effect of optic flow on steering behavior between groups. Although it is true that the traditional power analysis offered by GPower assumes equal sample sizes in its power calculation, an analysis of ”effective” sample size indicates that we have more subjects in both groups than required for 80% power on the interaction even without balancing the number of participants in the two group design through the collection of more CB data. For a two-group comparison, the ”effective” sample size^79^ is: $N_{\textrm{eff}} = \frac{4 n_1 n_2}{n_1 + n_2}$. Therefore, with $n_1 = 9$, and $n_2 = 21$, $N_{\textrm{eff}} \approx 25$. This suggests that our unbalanced design has about the same power as a balanced design with  12–13 participants per group, and this value exceeds the 7 per group criterion indicated by power analysis.

The results of this pooled, two-group analysis of mean distance from the inner road edge support the conclusions of the divided three-group analysis. Application of a mixed-ANOVA found that the optic flow density $\times$ group interaction did not reach significance after Greenhouse–Geisser correction ($F(1.39,39.02)=3.05$, $p=0.075$, $\eta _p^2=0.098$). The pooled data was also in agreement with the divided analysis in its finding of robust main effects of optic flow density, turn direction, turn radius, and an interaction between optic flow density and turn radius (optic flow density: $F(1.39, 39.02)=65.88$, $p<0.001$, $\eta _p^2=0.702$; turn direction: $F(1,28)=17.20$, $p<.001$, $\eta _p^2=0.380$; turn radius: $F(1.37,38.23)=5.63$, $p=0.015$, $\eta _p^2=0.168$; optic flow density $\times$ turn radius $F(3.21, 89.78)=6.17$, $p<0.001$, $\eta _p^2=0.181$).

A similar pooled analysis of lane-position variability also found no reliable difference between controls and CB participants, and no evidence that optic flow modulated variability differently across groups (group: $F(1,28)=3.54$, $p=0.070$, $\eta _p^2=0.112$; optic flow density $\times$ group: $F(1.951,54.616)=0.77$, $p=0.466$, $\eta _p^2=0.027$; turn radius $\times$ optic flow density $\times$ group: $F(2.615,73.215)=0.43$, $p=0.703$, $\eta _p^2=0.015$). Instead, lane-position variability showed strong main effects of turn radius and optic flow density, as well as a robust turn radius $\times$ optic flow density interaction (turn radius: $F(1.266,35.458)=132.64$, $p<0.001$, $\eta _p^2=0.826$; optic flow density: $F(1.951,54.616)=19.02$, $p<0.001$, $\eta _p^2=0.405$; turn radius $\times$ optic flow density: $F(2.615,73.215)=19.92$, $p<0.001$, $\eta _p^2=0.416$).

Taken together, the pooled analysis shows that collapsing CB participants into a single group does not strengthen evidence for CB-specific steering differences. Instead, the results support the interpretation that a simple CB vs control contrast risks obscuring differences driven by laterality-dependent effects of the loss. Stated more simply: analysis should respect the fact that CB-related effects on steering are heterogeneous.

### Gaze behavior

Figure 7 presents the distribution of within-trial azimuthal gaze positions observed across the middle 40% of each trial for each participant, separated by group. These data show that while most participants’ gaze was relatively symmetric about the car’s instantaneous heading (zero degrees), the centers of a few distributions were shifted slightly left or right, particularly within the CB groups. The relative position of these roughly symmetric distributions’ peaks were useful in revealing whether or not CB participants made compensatory scans into their blind field. If they did, the distribution of gaze for participants with CB would be shifted in the direction of their blind field. Visual inspection of the data does not lend strong support for this interpretation. In addition, the relative width of these distributions provided insight into the variability in the placement of gaze along the azimuth. These distributions showed left and right-sided CB participants to have relatively high levels of gaze position variability between members of their respective groups.

**Figure 8:**
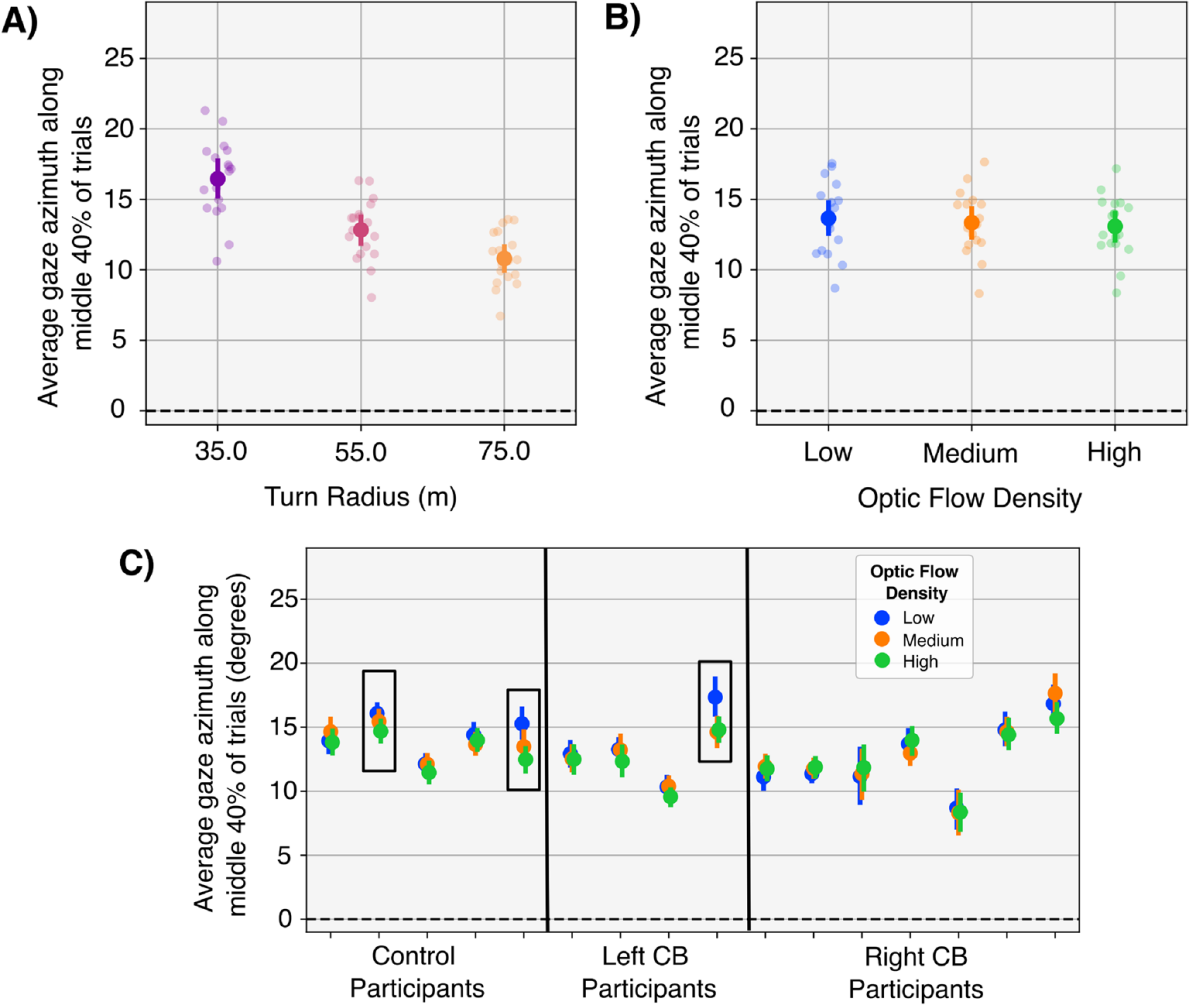
(**A**) Gaze azimuth across the central 40% of each trial for all participants and for each turn radius. All fixations on left turns were negated to ensure a normal distribution of gaze data prior to statistical analysis and plotting. Participants made more eccentric fixations on sharper turns ($p<0.001$). (**B**) Gaze azimuth across the central 40% of each trial for all participants and for each optic flow condition. The effect of optic flow density was significant, but (**C**) illustrates that the statistical significance is carried by only three participants. (**C**) Although the data in (**B**) as a function of optic flow was statistically significant, the significance was carried by only a few participants (black boxes).

To investigate whether participants modified gaze behavior in response to changes in optic flow, a mixed ANOVA was applied to azimuthal gaze position with group as a between-subjects variable and turn direction, turn radius, and optic flow density as within-subjects variables. The ANOVA revealed significant effects of turn radius and optic flow on gaze azimuth when averaged across the central 40% of the trial (Fig. 8A,B), but no differences between groups. All participants made more eccentric fixations on sharper turns $(F(1.22, 15.85) = 454$, $p < 0.001$, $\eta _p^2 = 0.972$, Fig. 8A). Although the effect of optic flow density on gaze azimuth was not apparent from visual inspection of Fig. 8B, the results of the ANOVA suggest the effect is statistically significant $(F(1.89, 24.60) = 5.72$, $p = 0.012$) with an effect size considered large by conventional standards ($\eta _p^2 = 0.306$). Further inspection of individual data (Fig. 8B) suggests that only a few participants carried the significant effect. To confirm this hypothesis, we recalculated the ANOVA after removing those three participants and indeed the effect of optic flow became insignificant ($p = 0.137$). The results imply that the effect of optic flow on gaze azimuth may be due to the small sample size.

**Figure 9:**
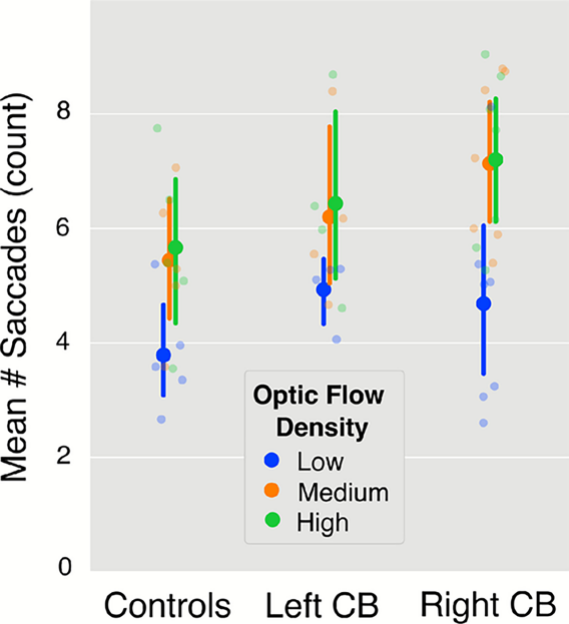
The average number of saccades executed during each turn, separated by optic flow density and participant group. Participants make fewer saccades on average when translational flow from self-motion was absent. The effect of group was not significant.

An additional analysis was conducted to test whether participants with CB made more saccades than those without CB. A difference in saccade rates could indicate a strategy used to compensate for the blind field and keep certain parts of the road within the intact visual field. This analysis was motivated by results of previous research that reported differences in gaze behavior between groups^10,44–48^ and the fact that no differences in gaze behavior had been identified thus far in the present study. Controls made an average of 4.96 (SD: 1.29) saccades per trial compared to 5.85 (SD: 1.22) for left-sided CB and 6.33 (SD: 1.31) for right-sided CB participants. The data is plotted in Fig. 9 as a function of optic flow and group. A mixed ANOVA with turn direction, turn radius, and optic flow density as independent variables found a statistically significant effect of optic flow density $(F(1.03,13.41) = 20.33$, $p = 0.001$, $\eta _p^2 = 0.558$), but no significant difference between groups. Although the effect of group appeared to be potentially significant in Fig. 9, the statistical power was too low to reveal significance.

## Discussion

In this study, we asked whether the presence of unilateral CB influences steering in a closed-loop virtual reality steering task where optic flow density was systematically manipulated. The study was designed to test the hypothesis that because CB might interfere with the processing of motion information, individuals with CB will place a higher weighting on more reliable alternative sources of information to guide the control of steering. In the context of our experiment, this would have been demonstrated as a weaker effect of manipulations of optic flow on steering behavior for participants with CB than for those without.

We found that all participant groups were affected by the manipulation of optic flow density. Analysis of mean lane position suggests that the control and Right CB group cut corners with greater magnitude when proximal flow from self-motion was visible (in the medium and high flow conditions). The finding that participants cut corners more on medium/high flow density trials is consistent with results from a previous experiment^58^ in which the authors interpreted the behavior as a minimized reliance on optic flow when the signal became more sparse or unreliable, as in the low flow density condition. This result suggests that the manipulation of optic flow was effective. Although the Left CB group did not demonstrate a within-group optic-flow effect on mean lane position, all groups demonstrated greater levels of maximum lane departure and increased lane-position variability in the low-flow condition. Although results indicated that average and maximum lane position varied as a function of an interaction between flow density and group (Fig. 5A,B), and this supports the hypothesis that CB affects motion processing, we advise that this result be accepted with some caution. Despite the significant interaction of group and flow density on mean lane position, post hoc analysis revealed no differences between groups and within flow density types (e.g., across all blue data points in Fig. 5). Further analysis of distance from the inner road edge showed the finding of significance to be carried by an attenuation of the effect of optic flow for the Left CB group that was not present in measures of maximum departure from the lane center or steering variability. Overall, we interpret this result as weak evidence in support of the central hypothesis that participants with CB demonstrate deficits to motion processing.

Several measures suggested that the location of visual impairment in CB patients may further impact steering behavior in the presence of optic flow manipulations. Correlations between the location of the visual deficit and steering behavior suggested that patients with a visual deficit on the left side of their visual field might be less sensitive to changes in optic flow, while those with right-sided deficits could be more sensitive. It is unlikely that these differences can be attributed to differences in visual information, for the reason that the structure of the visual environment was approximately symmetric about the vertical axis and participants navigated an equal number of left and right turns. Additionally, these results could not be explained by an analysis focused on the differences in the characteristics of the blind field or its onset between groups (e.g., age, blind field size, and the amount of sparing). For these reasons, it is possible that the relative insensitivity of left-sided CB participants to changes in optic flow is caused by differences in the way motion information is processed with a left-sided deficit, rather than differences in the stimulus. Although this result is unexpected, it is aligned with some current understanding of lateralization of motion processing in the brain. Several studies have found a greater role of the right than left hemisphere in the processing of motion information^80–82^. Given that the hemispheres of the primary visual cortex process information from the contralateral visual hemifield, it seems reasonable to speculate that damage to the right hemisphere early visual cortex, as in our left CB group, would have a greater effect on motion processing than damage to the same areas in the left hemisphere.

Our correlational analysis of blind field location and steering behavior also indicated that larger upper visual field deficits moderately reduced the steering differences between flow density conditions and larger lower visual deficits moderately increased them. Because the task environment was notably asymmetric about the horizontal meridian, these differences may be attributed to differences in the stimulus that arrived through the intact field. Particularly for the high flow density conditions, we speculate that an upper visual field deficit reduced the impact of trees on the magnitude of optic flow (see Fig. 1A), resulting in an overall decrease of flow impact on corner cutting. This theory is supported by the correlation results, which provided evidence that upper visual field deficits moderately correspond to reduced steering responses to changing optic flow.

The results of this study also revealed that steering by the control and right-sided CB groups differed with turn direction (left vs right), but steering by those with left-sided deficits did not (Fig. 4). Previously, it was found that left-sided CB participants were biased in lane position away from their blind field, and this was interpreted as a behavior intended to provide a margin of safety in the presence of moving obstacles like pedestrians and other cars^9^. This interpretation was not supported in the present study by post-hoc comparisons of between-group steering behavior within a given turn direction. It is likely that this result reflects differences in the task environments. The virtual task environment adopted by Bowers et al. (2010)^9^ was designed to capture the multi-task environment of real-world driving, which can be considered a coordinated execution of sub-tasks that include steering, speed control, visual search, and the monitoring of road signs, traffic, and more. Our study was instead designed to isolate the effect of specific variables on the sub-task of steering in the absence of moving obstacles. Our lack of evidence for CB bias in lane position away from their blind fields could be a result of the obstacle-free low-risk virtual environment we used. Overall, however, our results supported the fact that left-sided CB participants steer differently than controls and right-sided CBs as a function of turn direction. This finding supplements our conclusions of steering as a function of optic flow, where left-sided CB subjects also performed differently. Notably, both results are consistent with the outcomes of an on-road driving assessment study in which 4/6 left-sided CBs and 0/4 right-sided CBs failed the driving test on account of difficulty maintaining lane position and judging gaps^83^.

Interestingly, we did not find strong evidence that CB patients make compensatory scans towards their visual deficits to bring more of the road into their intact visual field, as has been found by several previous studies^10,44–48^. Individual gaze data (Fig. 7) indicates that while some participants may make more frequent scans towards the side of their blind fields on average, this is not the case for everyone, or even the majority, of CB drivers. The disassociation of gaze with steering behavior in regards to turn direction suggests that perhaps individuals with CB have no need to make compensatory scans in order to navigate successfully in this simplified virtual steering task. Additionally, as discussed previously, our task environment included no surprising obstacles, vehicles, or pedestrians and thus presented a low-risk navigation environment, potentially mitigating the need for compensatory scanning. Our follow-up analysis on saccade frequency between groups allowed us to investigate CB participants’ attempt to keep certain parts of the road within their intact visual field, but no statistical differences were found, which strengthens the interpretation that compensatory gaze strategies were unnecessary to complete this task.

Because glasses did not fit inside the HMD, a subset of participants performed this study under the presence of refractive blur. Previous studies have found that the drop in contrast and a loss in sensitivity to higher-frequency texture information in the presence of blur can reduce the influence of optic flow on heading judgments^84^. Accordingly, the color and appearance of the roadway and objects in the simulated environment were made to enhance contrast in the presence of blur with intention of mitigating this effect. There are several observations that suggest blur had little influence on steering behavior or the influence of optic flow in this task. The finding of a difference between the low and medium optic flow conditions indicates that contrast of the textured ground plane was sufficiently high to affect behavior even in the presence of refractive error. Similarly, Giguere et al. (2024)^58^ previously compared the behavior of younger and older drivers in a similar task and found that both groups reacted similarly to the manipulation despite the relatively larger number of participants with uncorrected refractive error in the older drivers group.

A general limitation of this study is in regards to the way CB blind fields are defined using Humphrey perimetry maps. While it is the most common method of assessing CB fields, Humphrey perimetry does so using a luminance detection task with a static stimulus, rather than a task requiring motion perception. Motion perception is often found within the CB field, at locations deemed ”blind” by static perimetry^85^. Since the present study investigated the use of motion information, the Humphrey-defined visual deficits may not have been the most appropriate way to measure CB fields. In addition, Humphrey perimetry maps span only 54$^\circ$ by 42$^\circ$, while the field of view of the HTC Vive Pro is much larger, approaching a 100$^\circ$ field of view. Thus, the visual capabilities of patients outside the view of the Humphrey visual fields, which may be critical for optic flow perception while steering, remain unknown.

What do the results of this study imply about whether CB represents a visual occlusion of motion information or a source of noise in motion processing? For some CB drivers, particularly those with right-sided CB, steering was quite similar to the behavior of drivers with normal vision. This indicates that CB could act as a minimally-impactful visual occlusion, rather than as a source of noise. It also suggests that optic flow incident on intact portions of the visual field could be sufficient for navigation. However, some participants with CB, and predominantly those with CB on the left side of their visual field, demonstrated a change in steering behavior relative to controls. This suggests that for these individuals, there may be some residual motion processing occurring in the blind field that complicates the processing of optic flow and affects steering. Ultimately, the variety of results emphasize the individual variability of CB deficits on steering. For a better test of the potential influence of individualized noise-corrupted motion information on steering, future work could take advantage of a more direct within-subject manipulation of visual information. For example, artificially masking CB visual deficits in a gaze-contingent manner while they steer could reveal whether these individuals utilize residual motion information from the blind field to navigate.

Although one might be motivated to generalize the findings of this study to an understanding of driving in the natural environment, there are significant differences between the current task and real-world driving that should discourage efforts to make direct comparisons. These include the lack of a visible dashboard, moving obstacles, oncoming traffic, real-world consequences for collision, the need to follow navigational directions, the use of an unnatural but high-contrast ground texture, and more. These departures from the natural context of driving were made specifically to provide a fair test of the central hypothesis that unilateral damage to early visual cortex affects the processing of optic flow that guides steering behavior. Had the data collected in the current task environment supported this evidence, it would have likely motivated a more careful consideration of the potential impact of this behavior in contexts that more closely resemble real-world driving.

Our results support the conclusion that only some participants with CB steer differently than those with normal vision in response to optic flow density and turn direction. We found evidence that the specific location of the blind field may influence the way participants steer in response to optic flow conditions. An impairment on the left visual field moderately reduced differences in corner-cutting between flow density conditions, while a right-sided deficit weakly increased the differences. Similarly, there was a moderate correlation between deficit location in the upper vs. lower visual field and steering. We found no evidence that CB subjects adopt different eye movement strategies than controls, as measured by gaze azimuth/elevation and saccade rate. Overall, CB steering and gaze behavior appeared to be remarkably unaffected despite the presence of visual deficits across large portions of the visual field.

## Data Availability

Access to compressed data files containing the behavioral metrics described in this manuscript can be found on GitHub at https://github.com/PerForm-Lab-RIT/RIT_CB_Steering_Data_2025. The repository includes de-identified data files for each participant as well as the analysis file that can be used to read in the data and visualize results.
